# Unveiling functional heterogeneity in pasteurized *Akkermansia muciniphila* identifies AKK2645 as a superior postbiotic for metabolic syndrome

**DOI:** 10.3389/fnut.2026.1908600

**Published:** 2026-08-06

**Authors:** Yaqiong Li, Wenfang Song, Xiaoli Liu, Jie Du, Linbo Li, Fan Wang, Kaige Zhang

**Affiliations:** 1 Hunan Province Directly Affiliated TCM Hospital, Zhuzhou, China; 2 Langfang Hotgen Biotechnology Inc., Langfang, China; 3 Department of Health Examination Center, Shaanxi Provincial People’s Hospital, Xi’an, China; 4 Health Management Center, The Second People’s Hospital of Hunan Province, Changsha, China; 5 The Clinical Laboratory, The First Affiliated Hospital of Xi’an Jiaotong University, Xi’an, China

**Keywords:** AKK2645, *Akkermansia muciniphila*, functional heterogeneity, metabolic syndrome, postbiotic

## Abstract

Metabolic syndrome (MetS) represents an escalating global health burden, underscoring an urgent need for innovative microbiome-targeted therapeutics. This investigation involved a systematic functional evaluation of 42 pasteurized *Akkermansia muciniphila* strains, providing the first direct evidence of significant differences among strains in their ability to alleviate MetS. Through systematic screening using cell and mouse models, we identified strain *Akkermansia muciniphila* YGMCC2645 (AKK2645) emerged as the lead candidate. Furthermore, it demonstrated superior and reproducible efficacy in attenuating diet-induced obesity, restoring glucose homeostasis, and ameliorating hepatic steatosis. AKK2645 uniquely modulated host immunity, decreasing pro-inflammatory cytokines (IL-6, IL-1β) and increasing anti-inflammatory markers (*Il4*, *Il10*, and *Foxp3*) in both adipose and hepatic tissues. It also favorably altered gut microbiota (increasing *Akkermansia*, L*actobacillus*,and *Bifidobacterium*) and activated intestinal GLP-1 signaling. Collectively, these findings establish strain-specific functional heterogeneity as a fundamental principle governing postbiotic efficacy and identify AKK2645 as a high-potency, precision postbiotic for MetS intervention.

## 1 Introduction

Metabolic syndrome (MetS) encompasses a cluster of interconnected metabolic derangements, including central adiposity, impaired insulin signaling, atherogenic dyslipidemia, and elevated arterial pressure, which collectively increase the risk of cardiovascular morbidity and type 2 diabetes mellitus (1). Its prevalence continues to rise unabated worldwide, posing an increasingly formidable public health imperative. Beyond conventional genetic and lifestyle determinants, dysbiosis of the gut microbiota and the consequent state of chronic low-grade systemic inflammation are now recognized as pivotal drivers in both the pathogenesis and progression of MetS (2, 3). These insights have positioned targeted modulation of the intestinal microbial ecosystem as a strategically promising frontier for MetS management.

Among gut-resident commensals, *Akkermansia muciniphila* (AKK) has garnered considerable scientific attention, owing to its robust inverse correlation with host metabolic dysfunction, its relative abundance is consistently diminished in individuals with obesity, insulin resistance, and MetS (4, 5). Notably, heat-inactivated preparations of this mucin-degrading bacterium have emerged as particularly promising therapeutic modalities, conferring robust metabolic benefits in diverse animal models of metabolic dysfunction (6–8). These preclinical observations have been successfully translated to clinical settings, where supplementation with heat-inactivated AKK safely enhanced insulin sensitivity and ameliorated lipid profiles in overweight and obese volunteers (9, 10). Such preparations, classified as postbiotics, exert health benefits through non-viable microorganisms or their bioactive components (11).

Compared with live biotherapeutics, postbiotics offer defined safety advantages, improved scalability, and greater physicochemical stability, while eliminating risks associated with antibiotic resistance gene transfer—features that collectively facilitate clinical translation (3, 12). Although intraspecies functional heterogeneity is well documented in probiotic research (13, 14), postbiotic frameworks have largely overlooked strain dependency (15, 16). Studies on pasteurized *Akkermansia muciniphila* (pAKK) have relied almost exclusively on single reference strains, most notably ATCC BAA-835 (17–19), leaving the extent and reproducibility of inter-strain variation unresolved. Whether phylogenetically distinct pAKK strains differ in their capacity to ameliorate MetS therefore remains an open and clinically relevant question.

To address this, we conducted a parallel functional screen of pAKK strains. Candidate strains were sequentially evaluated using a tiered pipeline, beginning with *in vitro* assays for anti-lipidogenic and anti-inflammatory activities, followed by *in vivo* validation in a HFD-induced murine model of MetS. This approach enabled us to define the functional heterogeneity of pAKK strains, identify *Akkermansia muciniphila* YGMCC2645 (AKK2645) as a lead candidate, and dissect the multimodal mechanisms underlying its enhanced efficacy. These findings establish strain specificity as a key determinant of postbiotic performance and identify AKK2645 as a promising therapeutic candidate for MetS.

## 2 Materials and methods

### 2.1 Bacterial isolation, cultivation, RNA extraction, inactivation, and P9 quantification

The 42 strains of AKK were isolated from fecal samples of healthy Chinese donors using an established protocol (20). These strains have been deposited in the Microbial Culture Collection Center of Yujing Pharmaceutical Co., Ltd. (Beijing, China). The reference strain ATCC BAA-835 was obtained from the American Type Culture Collection (ATCC). All strains were cultivated under standard anaerobic conditions to mid-log phase, with cell densities normalized across strains. Total RNA was isolated using the TransZol Up Plus RNA Kit (TransGen Biotech, China) per the manufacturer’s protocol. First-strand cDNA was generated from the extracted RNA with the PrimeScript RT Reagent Kit (Takara, Japan) and stored at −80 °C.

All strains were subjected to cultivation, pasteurization, viable cell residue detection, and preservation according to standard procedures, the pasteurization of AKK was conducted at 70 °C for 30 min to obtain the pAKK (21, 22). The strains were lyophilized under the following parameters: a condenser temperature of −80 °C, chamber pressure of 0.63 mbar, and a two-stage drying protocol consisting of 12 h at 0 °C followed by 3 h at 30 °C. The resultant powders were stored at room temperature in vacuum-sealed containers until further use. Viability assays demonstrated that bacterial counts in the final pAKK preparations fell below the detection threshold (<10 CFU/g). Storage stability results show that, under the recommended storage conditions, the bioactivity and total fluorescence units (TFU) count of the lyophilized powder remained stable throughout the testing period. The pAKK underwent serial dilution and subsequent staining with SYTO™24 (0.1 mM, Invitrogen, USA) at 37 °C for 15 min in a dark environment. The stained samples were then subjected to analysis utilizing a BD Accuri™ C6 Plus flow cytometer (BD Biosciences, USA), which had been calibrated with BD CS&T quality control beads. Bacterial events were delineated based on FSC/SSC profiles, and SYTO™24-positive particles were enumerated as intact cells. The pAKK concentration was normalized to TFU per mL or per gram of powder (23). Quantification of P9 protein levels was performed using an ELISA kit, a generous gift from Thankcome Biological Science and Technology (SuZhou) Co., Ltd. (China), following the manufacturer’s specified protocol.

### 2.2 *In vitro* cell-based assays

HepG2 cells (Wuhan Puri Life Science and Technology Co., Ltd., China) were maintained in minimal essential medium (MEM) (Wuhan Puri Life Science and Technology Co., Ltd., China) supplemented with 1 mM oleic acid (OA) (Sigma-Aldrich, USA) for 5 h to induce lipid accumulation (24). Following this, cells were treated with 42 pAKK powder, including the reference strain ATCC BAA-835, at a titer of 1 × 10^8^ TFU/mL in MEM medium for 5 h. Experimental groups comprised a control group (Con) and an OA-induced model group (OA). Intracellular lipid deposition was evaluated morphologically through Oil Red O (Beijing Solarbio Science & Technology Co., Ltd., China) staining, and triglyceride (TG) content was measured using a commercial TG assay kit (Nanjing Jiancheng Bioengineering Institute, China).

For HT-29 cells (Wuhan Puri Life Science and Technology Co., China), pre-incubation with pAKK strains (1 × 10^8^ TFU/mL) was performed for 6 h. This was followed by stimulation with lipopolysaccharide (LPS, 1 μg/mL) (Beijing Solarbio Science & Technology Co., Ltd., China) for 6 h to induce an inflammatory response (25). The pAKK BAA-835 powder was used as the reference strain in each independent experiment. Cell culture supernatants were then gathered, and the concentrations of interleukin-8 (IL-8) and interleukin-1β (IL-1β) were quantified using the MQ60 plus system (Hotgen, China).

### 2.3 Animal experiments

The study received approval from the Institutional Animal Care Committee of the Beijing Sungen Biomedical Technology Co., Ltd. (Approval Number: IACUC-SG-AM-YU-23001), and protocols strictly followed established methodologies (26). Eight-week-old male C57BL/6 J mice were acclimated for 3 days before being randomly allocated into 10 experimental groups (*n* = 10): one normal diet (ND) control group, one HFD model group, and 8 HFD + pAKK intervention groups. To induce MetS, mice in the HFD groups were fed a 60% kcal HFD (Xietong Bio, China), while ND controls received a standard chow diet. Starting at week 0, the pAKK intervention groups were administered 100 μL of their respective pAKK suspension daily via oral gavage (approximately 1 × 10^8^ TFU/mouse/day). The ND and HFD control groups received an equal volume of phosphate-buffered saline (PBS). The intervention period lasted for 8 weeks, with body weight measurements taken weekly.

Approximately 12 h before the intervention concluded, we measured fasting blood glucose and conducted intraperitoneal glucose tolerance tests (IPGTT) using a 2 g/kg glucose dose, subsequently calculating the area under the curve (AUC). Following sacrifice, we collected serum, liver, white adipose tissue (WAT) depots, epididymal white adipose tissue (eWAT) and inguinal white adipose tissue (iWAT), brown adipose tissue (BAT), and fecal samples. Serum TG and total cholesterol (TC) levels were quantified via standard biochemical assays. Tissue samples were fixed, embedded in paraffin, sectioned, and stained with hematoxylin and eosin (H&E) for histopathological examination (27). Certain tissues were immediately frozen at −80 °C for future molecular analyses.

### 2.4 RNA extraction and quantitative real-time PCR

RNA extraction and quantitative real-time quantitative PCR (qPCR) were conducted following the methodology previously detailed by He et al. (28). Total RNA was isolated from liver, eWAT, iWAT, BAT, and ileum tissues using the TransZol Up Plus RNA Kit (TransGen Biotech, China) according to the manufacturer’s protocol. For adipose tissue samples, an additional purification step was included: Trizol-lysed samples were centrifuged at 12,000 rpm for 10 min at 4 °C to remove residual lipids and improve RNA purity. First-strand cDNA was synthesized from 1 μg of total RNA using the PrimeScript RT Reagent Kit with gDNA Eraser (Takara, Japan) following the manufacturer’s instructions. qPCR was performed using the Rapid Fluorescence PCR Kit (Tiangen, China) on a LightCycler® 480 Real-Time PCR System (Roche, Switzerland). The thermal cycling protocol was as follows: initial denaturation at 95 °C for 30 s, followed by 40 cycles of 95 °C for 5 s and 60 °C for 30 s. *β*-actin was used as the internal reference gene. Relative gene expression levels were calculated using the 2^−ΔΔCt^ method. All primers used in this study are listed in Supplementary Table S1.

Total microbial genomic DNA was extracted from fecal samples using the PF Mag-Bind Stool DNA Kit (Omega Bio-tek, GA, USA). qPCR was used to determine the absolute concentration of beneficial bacteria in the fecal samples (29). The primer sequences were obtained from (30), see Supplementary Table 1 for details. The qPCR amplification conditions for detecting beneficial bacteria are 30 s at 95 °C, 5 s at 95 °C, and 40 s at 60 °C. Plasmids cloned by amplifying the gene sequences of model strains of beneficial bacteria were used as absolute quantification standards, and a standard curve was established using serial dilutions. The standard curve was plotted based on the copy numbers of the standards and their C_t_ values. The C_t_ values of the test samples were plotted on the standard curve to calculate the copy numbers of the target gene in the test samples. Each assay was repeated three times.

### 2.5 16S rRNA gene sequencing and gut microbiota analysis

The V3-V4 hypervariable region of the bacterial 16S rRNA gene was amplified with universal primers 338F (5′-ACTCCTACGGGAGGCAGCAG-3′) and 806R (5′-GGACTACHVGGGTWTCTAAT-3′). The PCR reaction was performed in a total volume of 25 μL containing 12.5 μL of 2 × Phusion High-Fidelity PCR Master Mix (New England Biolabs, USA), 0.2 μM of each primer, and 10 ng of template DNA. The amplification protocol consisted of an initial denaturation at 98 °C for 30 s, 30 cycles of denaturation at 98 °C for 10 s, annealing at 54 °C for 30 s, and extension at 72 °C for 30 s, and a final extension at 72 °C for 5 min. The resulting amplicons were sequenced on the Illumina MiSeq PE300 platform (Illumina, USA) at Majorbio Bio-Technology (Shanghai, China). Sequencing data were processed and analyzed to assess differences in microbial community structure among experimental groups. Functional predictions of microbial communities were performed using PICRUSt2 (version 2.2.0). The raw sequencing data have been deposited in the NCBI Sequence Read Archive (SRA) under BioProject accession number PRJNA1476887.

### 2.6 Protein structure visualization

The structure of the protein was obtained from the Protein Data Bank (PDB ID: 6LAD). Full-length models of Amuc_1100 and P9 (Amuc_1631) were generated by AlphaFold3. Structural representations were prepared with PyMOL 2.5.

### 2.7 Statistical analysis

All data are presented as mean ± standard deviation (SD). Statistical analyses were performed using GraphPad Prism 8 (GraphPad Software Inc., San Diego, CA, USA). Each experiment was conducted in triplicate and independently repeated at least twice. Comparisons between groups were made using unpaired and paired Student’s t-tests, depending on the experimental design. For comparisons involving multiple treatment groups against a single control group, one-way analysis of variance (ANOVA) was first applied to determine overall significance, followed by Dunnett’s post-hoc test for pairwise comparisons against the HFD control group. A *p*-value < 0.05 was considered statistically significant. Statistical significance was denoted as follows: ^*^p < 0.05, ^**^p < 0.01, ^***^p < 0.001, ^****^p < 0.001 versus HFD group.

## 3 Results

### 3.1 Functional heterogeneity among pAKK strains in metabolic regulation

**Figure 1:**
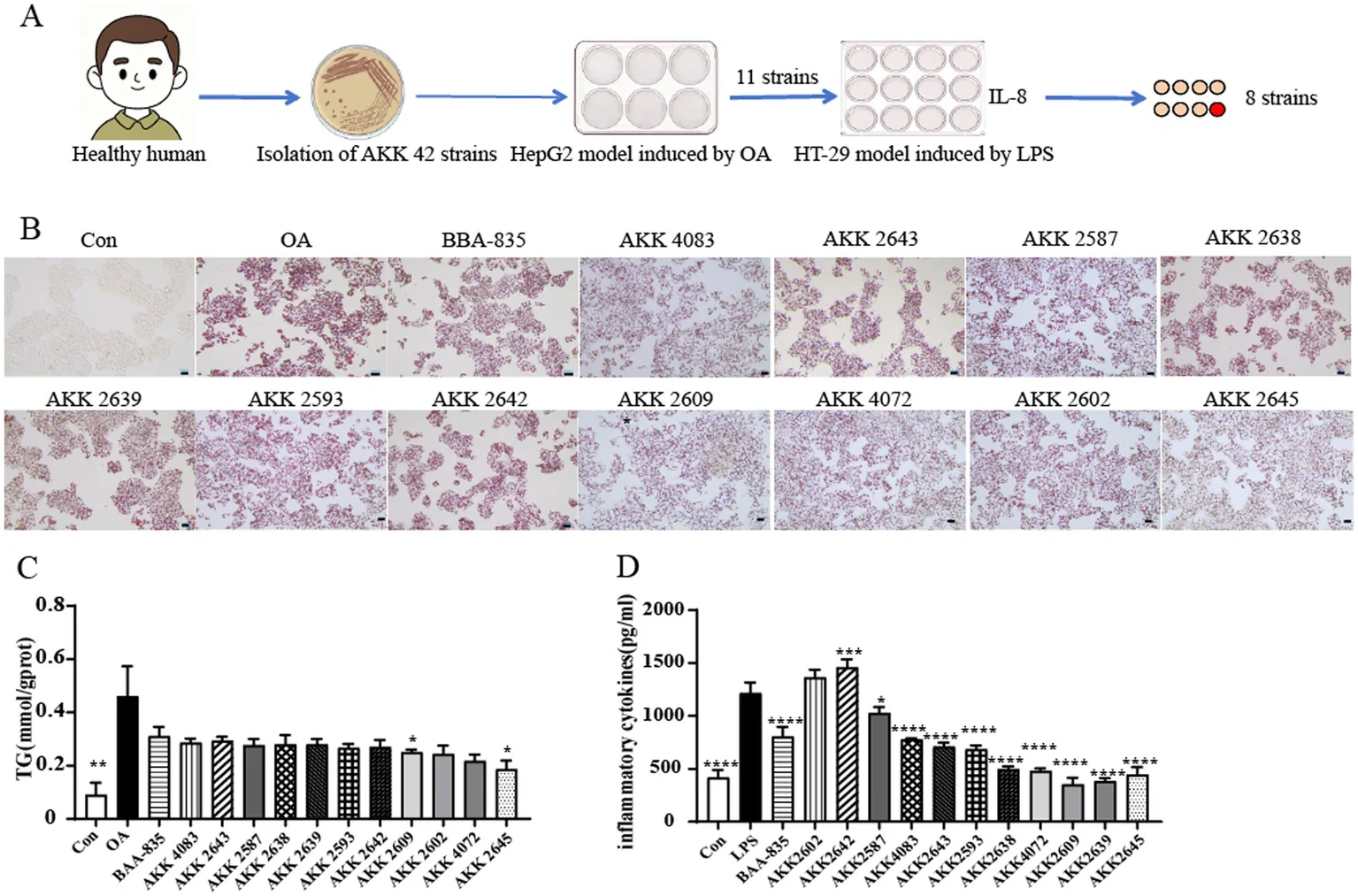
*In vitro* screening identifies pAKK strains with anti-adipogenic and anti-inflammatory bioactivities. **(A)** Schematic overview of the *in vitro* screening workflow. **(B)** Representative Oil Red O staining of HepG2 cells (20 ×, scale bar, 100 μm). **(C)** Quantification of intracellular TG content in HepG2 cells. **(D)** Protein levels of IL-8 in HT-29 cell culture supernatants. Data are presented as means ± *SD*. Statistical significance: *p < 0.05, ^**^p < 0.01, ^***^p < 0.001, ^****^p < 0.0001.

To identify promising postbiotic candidates, 42 pAKK strains were initially screened for their capacity to alleviate metabolic and inflammatory stress in cellular models (Figure 1A). In an oleic acid-induced HepG2 hepatocyte steatosis model, 34 strains significantly reduced intracellular TG content. Among these, 11 strains, including AKK2645, outperformed the reference strain ATCC BAA-835, with AKK2645 exhibiting the highest TG inhibition rate (59.24%) (Figures 1B,C; Supplementary Table S2). In a parallel assay using LPS-stimulated HT-29 intestinal epithelial cells, the results showed that 8 of these strains exhibited a higher inhibition rate of pro-inflammatory chemokine IL-8 secretion than ATCC BAA-835 (31.89%) (Figure 1D). The eight selected strains demonstrated superior *in vitro* anti-lipidogenic and anti-inflammatory activities compared to the BAA-835 reference strain. *In vitro* analyses revealed that these eight strains conferred augmented anti-lipidogenic and anti-inflammatory capacities relative to the reference strain ATCC BAA-835. Consequently, these eight candidate strains were selected for subsequent *in vivo* evaluation.

### 3.2 Superior efficacy of pasteurized AKK2645 in attenuating diet-induced obesity and dyslipidemia

**Figure 2:**
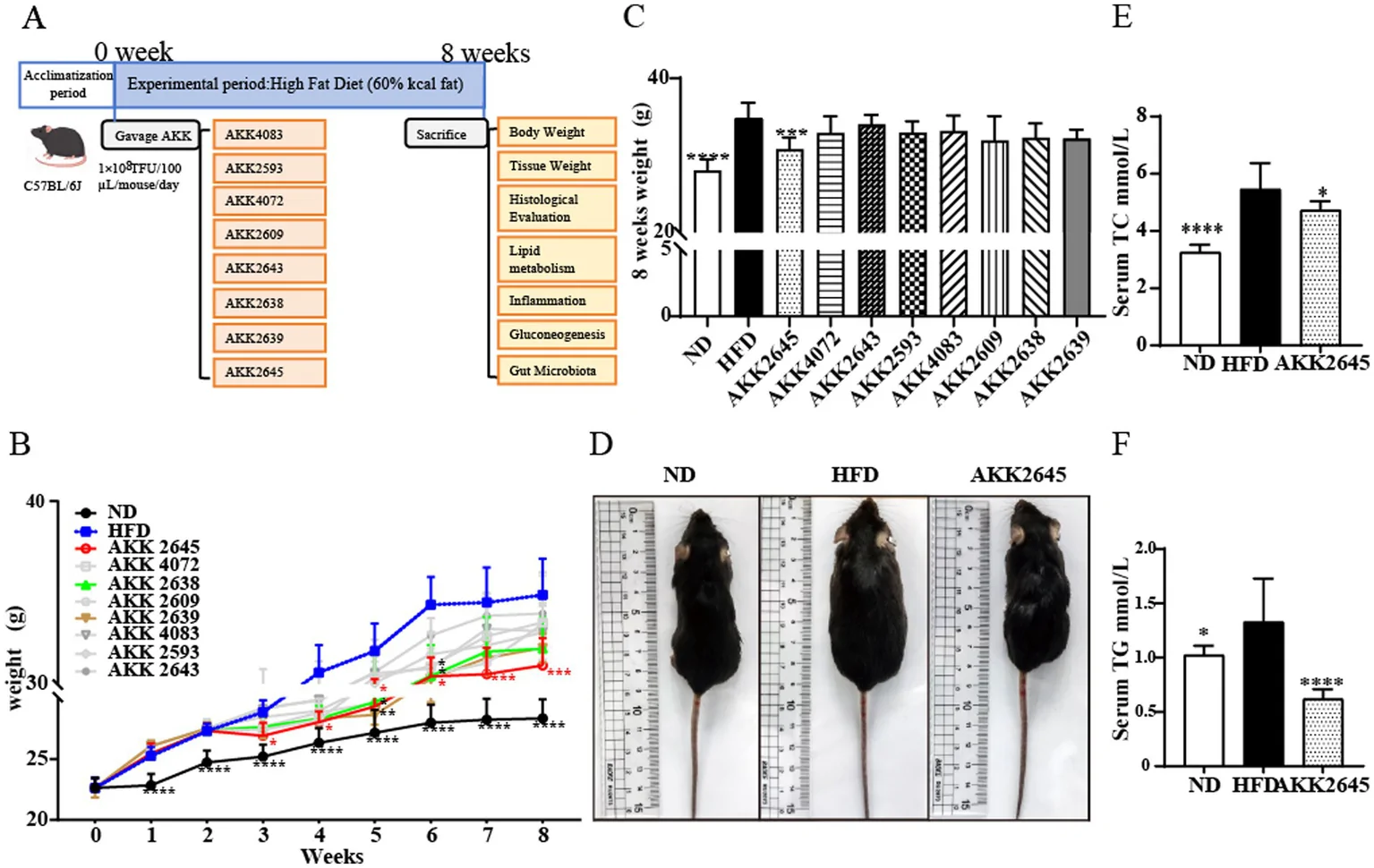
pAKK strains inhibit HFD-induced obesity in mice. **(A)** Experimental timeline. **(B)** Weekly body weight trajectories. **(C)** Body weight at week 8. **(D)** Representative body morphology at week 8. **(E)** Serum TC levels. **(F)** Serum TG levels. Data are presented as means ± SD. Statistical significance: ^*^ *p* < 0.05, ^**^p < 0.01, ^***^p < 0.001, ^****^p < 0.0001.

The anti-obesity efficacy of the eight candidate pAKK strains was evaluated in HFD-fed mice (Figure 2A). Following an 8-week intervention, pasteurized AKK2645 demonstrated the most pronounced effect in attenuating HFD-induced body weight gain (Figures 2B–D). Consistently, administration of pasteurized AKK2645 most potently reversed HFD-induced elevations in serum TC and TG (Figures 2E,F). These results establish AKK2645 as the most efficacious candidate for mitigating HFD-induced obesity and dyslipidemia.

### 3.3 Pasteurized AKK2645 mitigates hepatic steatosis and restores adipose tissue function

**Figure 3:**
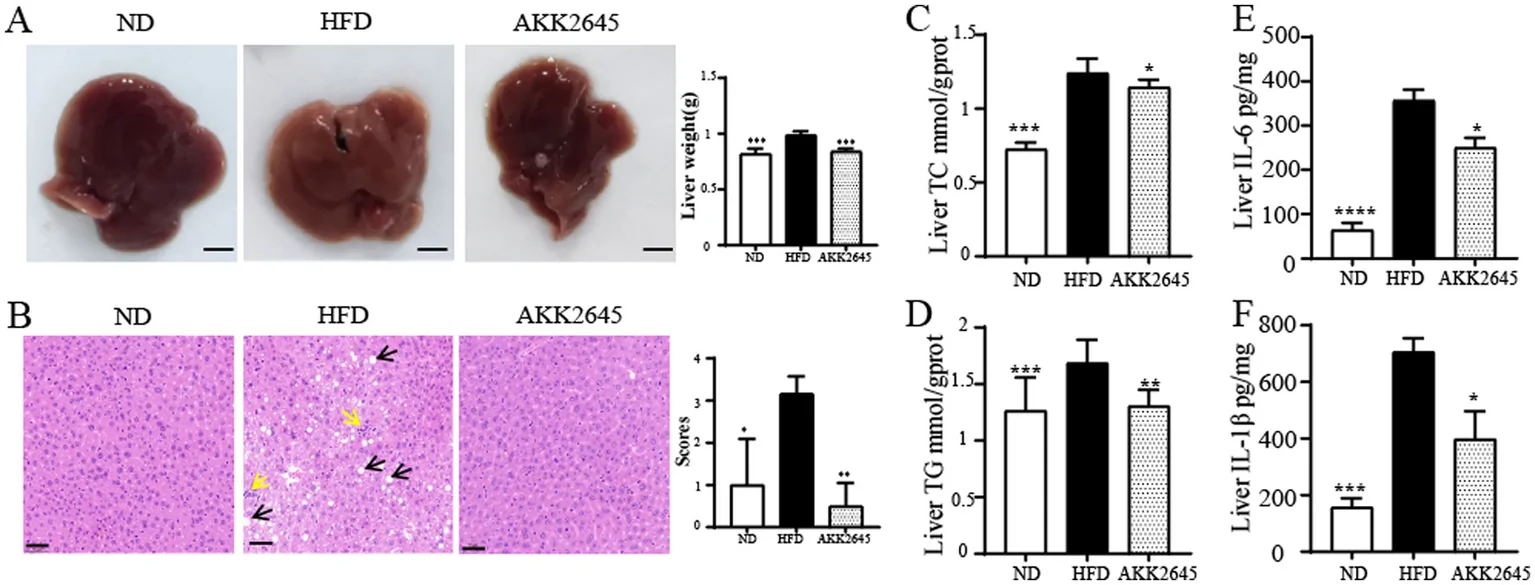
Pasteurized AKK2645 suppresses HFD-induced hepatic steatosis in mice. **(A)** Representative liver images (left) and liver weight (right) at week 8. **(B)** Representative H&E-stained liver sections (left, 20 ×, scale bar, 50 μm, black arrows indicate steatosis, yellow arrows indicate inflammatory infiltration) and quantitative histopathological scores (right). **(C)** Hepatic TC content. **(D)** Hepatic TG content. **(E)** Hepatic IL-6 protein levels. **(F)** Hepatic IL-1β protein levels. Data are presented as means ± SD. Statistical significance: ^*^p < 0.05, ^**^p < 0.01, ^***^p < 0.001.

**Figure 4:**
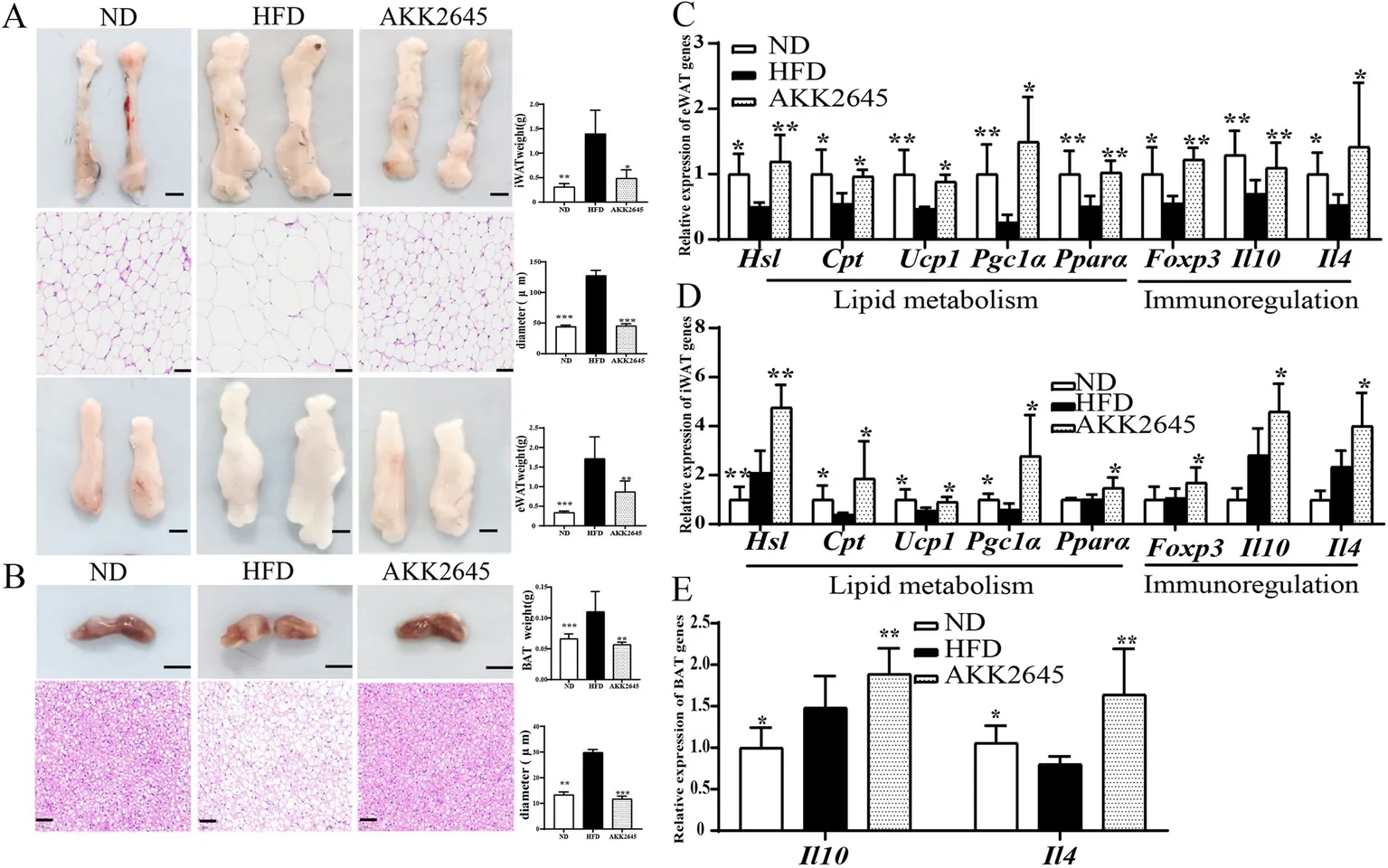
Pasteurized AKK2645 promotes lipolysis and anti-inflammatory cytokine production in adipose tissues. **(A)** Representative iWAT images (top left, scale bar, 5 mm), iWAT weight (top right), representative H&E-stained iWAT sections (middle left, 20 ×, scale bar, 50 μm), and quantitative histopathological scores (middle right), representative eWAT images (bottom left, scale bar, 5 mm) and eWAT weight (bottom right). **(B)** Representative BAT images (top left, scale bar, 5 mm), BAT weight (top right), representative H&E-stained BAT sections (bottom left, 20 ×, scale bar, 50 μm), and quantitative histopathological scores (bottom right). **(C–E)** Relative mRNA expression of metabolic and inflammatory regulators in iWAT, eWAT, and BAT, assessed by qPCR. Data are presented as means ± SD. Statistical significance: ^*^p < 0.05, ^**^p < 0.01, ^***^p < 0.001, ^****^p < 0.001.

Further characterization revealed that AKK2645 robustly improved metabolic organ pathology. In the liver, it markedly attenuated HFD-induced steatosis, inflammatory cell infiltration, and hepatomegaly (Figures 3A,B), concomitant with reduced hepatic lipid content and downregulated expression of pro-inflammatory cytokines IL-6 and IL-1*β* (Figures 3C–F). In WAT, AKK2645 intervention reduced depot mass and adipocyte size (Figure 4A). Mechanistically, the treatment upregulated gene expression involved in lipolysis (hormone-sensitive lipase, *Hsl*), fatty acid β-oxidation (peroxisome proliferator-activated receptor-*α*, *Ppara*, carnitine palmitoyltransferase 1A, *Cpt1a*), and thermogenesis (peroxisome proliferator-activated receptor *γ* coactivator 1-α, *Pgp1α*, uncoupling protein 1, *Ucp1*). Concurrently, it elevated mRNA levels of anti-inflammatory mediators (*Il4*, I*l10*, and *Foxp3*) (Figures 4C,D). Furthermore, AKK2645 effectively countered HFD-induced whitening of BAT, as evidenced by reduced lipid accumulation, increased tissue density, and enhanced local expression of *Il4* and *Il10* (Figures 4B,E). These data indicate that AKK2645 effectively ameliorates the metabolic and inflammatory milieu in key metabolic tissues.

### 3.4 Pasteurized AKK2645 exhibits superior efficacy in glucose homeostasis regulation

**Figure 5:**
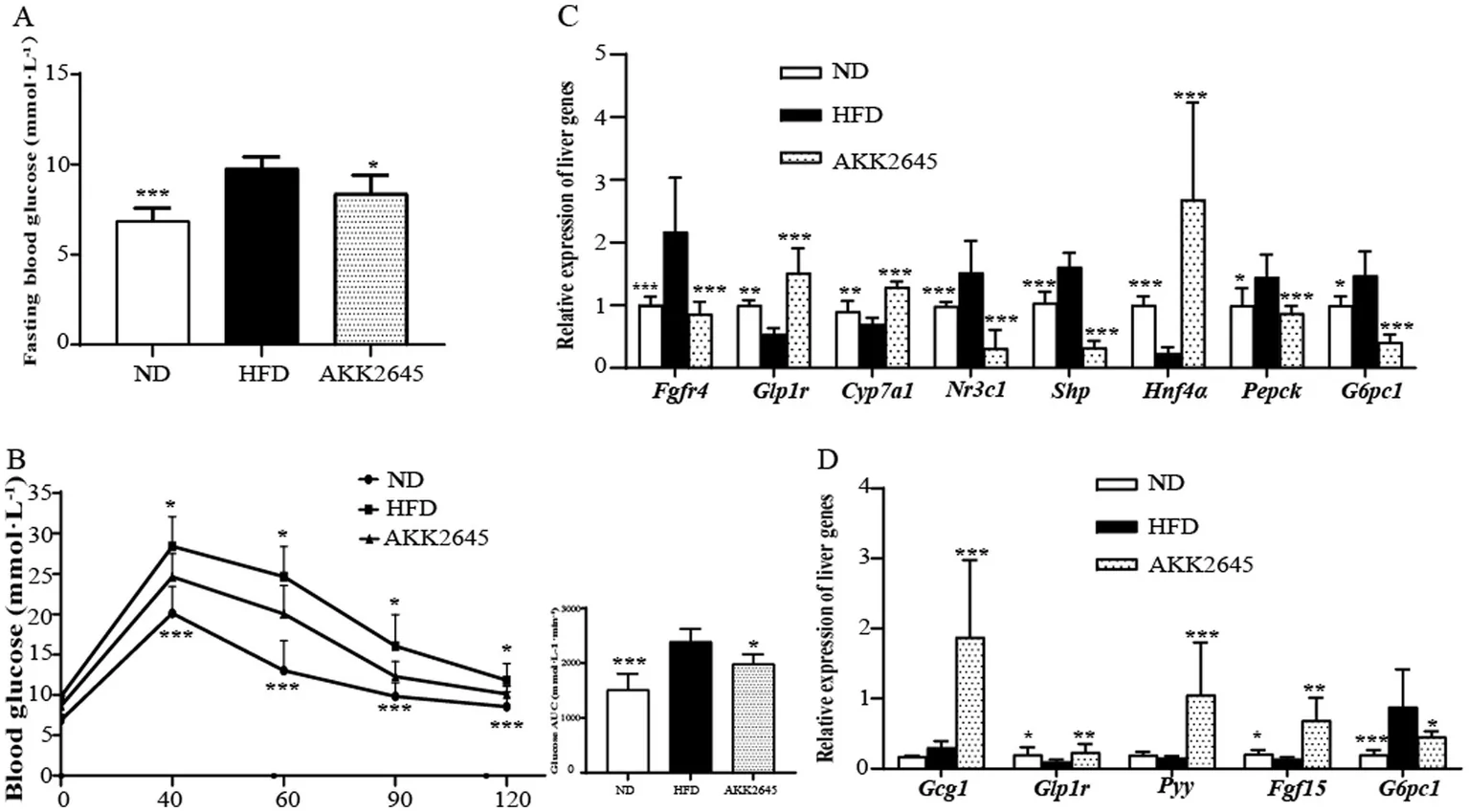
Pasteurized AKK2645 improves glucose homeostasis. **(A)** Fasting blood glucose levels. **(B)** Glucose tolerance test (left) and corresponding AUC (right). **(C,D)** Relative mRNA expression of metabolic regulators in the ileum and liver, assessed by qPCR. Data are presented as means ± SD. Statistical significance: ^*^p < 0.05, ^**^p < 0.01, ^***^p < 0.001.

Pasteurized AKK2645 demonstrated the most pronounced effects on glucose metabolism. It significantly lowered fasting blood glucose levels and enhanced glucose clearance during IPGTT, yielding a significantly reduced AUC compared to HFD group (Figures 5A,B). In the liver, treatment upregulated hepatic nuclear factor 4α (*Hnf4a*) and glucagon-like peptide-1 receptor (*Glp1r*) expression, while downregulating the glucocorticoid receptor (*Nr3c1*) and key gluconeogenic enzymes (phosphoenolpyruvate carboxykinase, *Pck1*, glucose-6-phosphatase catalytic subunit, *G6pc1*), consistent with suppression of hepatic glucose output (Figure 5C). In the ileum, AKK2645 uniquely and significantly elevated expression of GLP-1 and its downstream targets *glp1r* and peptide YY (*Pyy*), while suppressing ileum *G6pc1* expression. Additionally, the strain upregulated ileal fibroblast growth factor 15 (*Fgf15*) and correspondingly downregulated hepatic FGF receptor 4 (*Fgfr4*) and small heterodimer partner (*Shp*). It also increased hepatic cholesterol 7-alpha hydroxylase (*Cyp7a1*) expression (Figure 5D). This coordinated modulation of ileum and hepatic signaling pathways indicates a direct influence on the gut-liver metabolic axis. These convergent molecular adaptations highlight the exceptional capacity of AKK2645 to restore systemic glucose homeostasis through targeted intestinal-hepatic crosstalk.

### 3.5 Pasteurized AKK2645 distinctly remodels the gut microbiota

**Figure 6:**
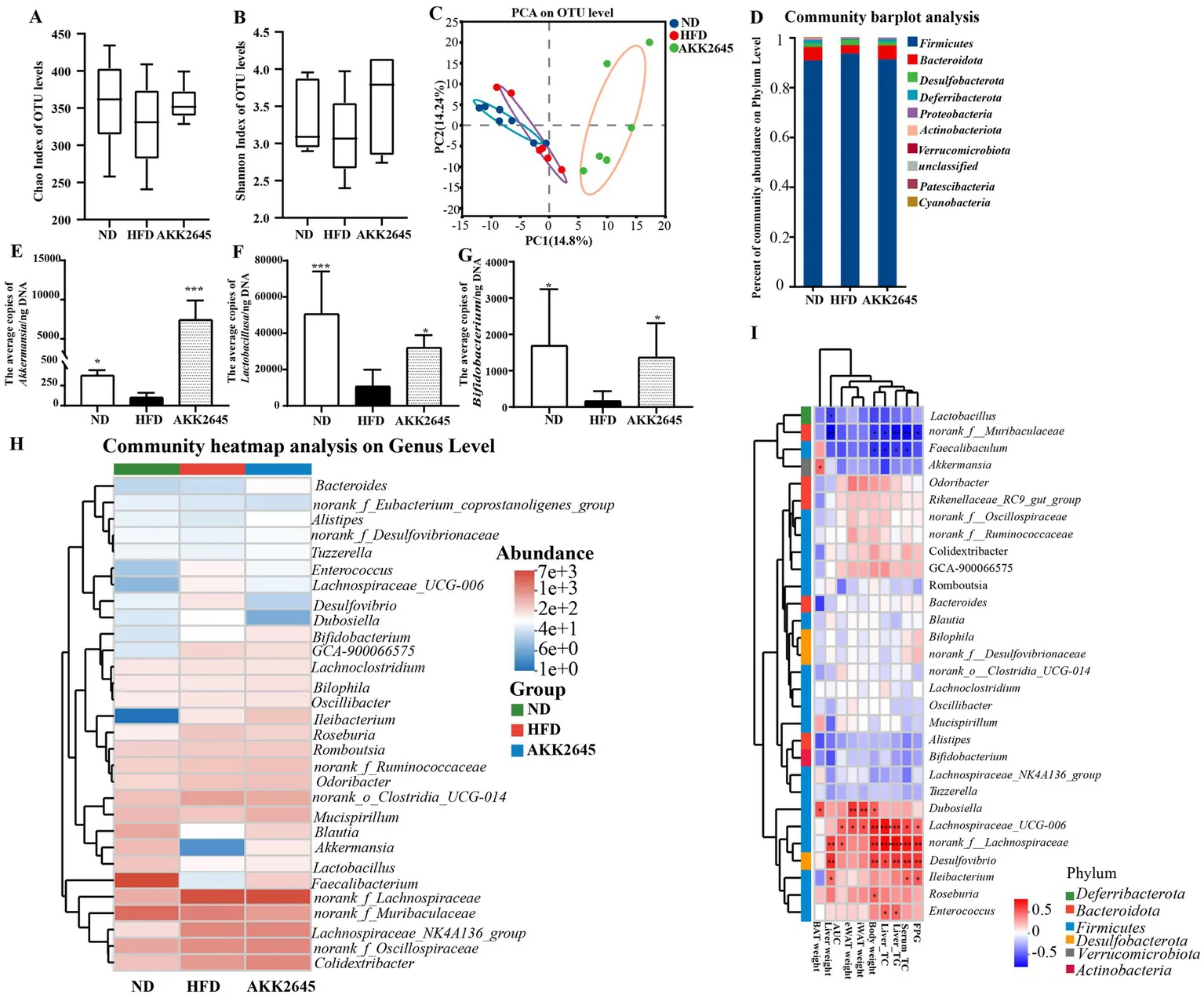
Modulation of gut microbiota by pasteurized AKK2645 in obese mice. **(A)** Chao1 index. **(B)** Shannon index. **(C)** Principal component analysis of gut microbial communities. **(D)** Relative abundance at the phylum level. **(E–G)** The absolute abundances of *Akkermansia*, *Lactobacillus* and *Bifidobacterium* in mouse feces were determined using qPCR. **(H)** Relative abundance at the genus level. **(I)** Correlation analysis between gut microbiota at the genus level and obesity-related parameters. Data are presented as means ± SD. Statistical significance: ^*^p < 0.05, ^**^p < 0.01, ^***^p < 0.001.

Analysis of the gut microbiota revealed that pasteurized AKK2645 intervention uniquely reshaped the microbial community disrupted by HFD. It enhanced *α*-diversity and altered *β*-diversity (Figures 6A–C). Analysis at the phylum level revealed an expansion of *Actinobacteriota*, *Verrucomicrobiota*—the phylum containing *Akkermansia*—and *Bacteroidota*, concurrent with a contraction of *Firmicutes* and *Desulfobacterota* (Figure 6D). Pasteurized AKK 2645 intervention significantly increased the abundance of *Akkermansia*, *Lactobacillus*, and *Bifidobacterium* in the feces of HFD mice (Figures 6E–G). At the genus level, AKK2645 increased the relative abundance of beneficial genera, including *Akkermansia*, *Lactobacillus*, and *Bifidobacterium*, while suppressing potentially detrimental genera, such as *Enterococcus*, *Dubosiella*, *Desulfovibrio*, and *Lachnospiraceae_UCG-006* (Figure 6H). Correlation analysis further established that the microbial consortia enriched by AKK2645—specifically *Faecalibacterium*, *Akkermansia*, and *Lactobacillus*—were negatively correlated with key obesity and glucolipid metabolic markers. Notably, *Faecalibaculum* and *Akkermansia* also showed positive correlations with BAT weight, suggesting that microbiota remodeling contributes to improved systemic energy balance (Figure 6I). This beneficial and distinct modulation of the gut ecosystem underscores a key mechanism contributing to the postbiotic’s superior efficacy.

### 3.6 Differential expression of P9 and Amuc_1100 across strains

**Figure 7:**
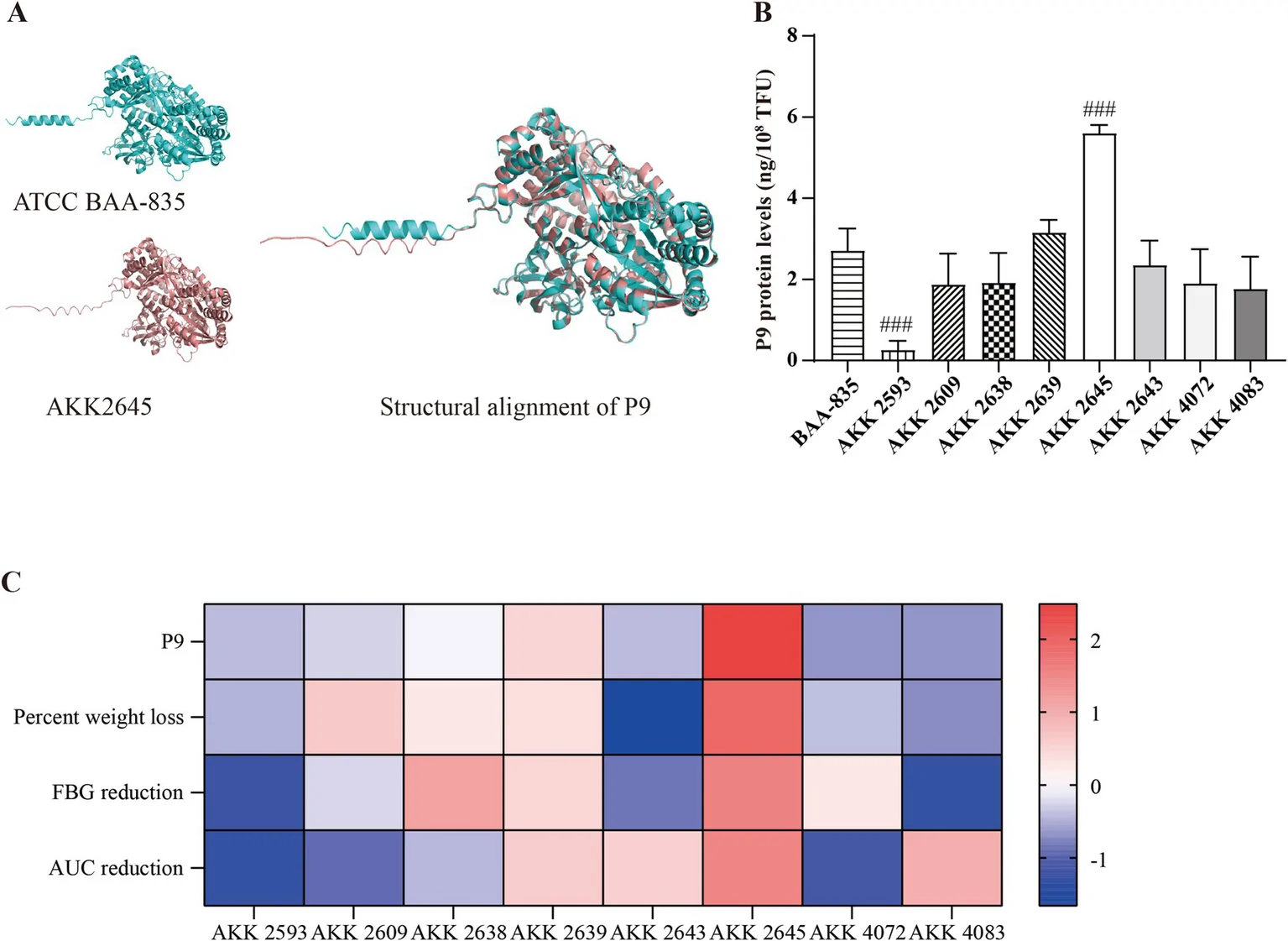
Sequence variation, expression, and phenotypic correlation of the P9 effector protein. **(A)** Schematic representation of amino acid mutations in P9 between the AKK2645 strain and reference strains. **(B)** Protein levels of P9 across different strains. **(C)** Heatmap depicting correlations between P9 protein levels and key metabolic phenotype parameters. Data are presented as means ± SD. ^#^*p* < 0.05, ^##^*p* < 0.01, ^###^*p* < 0.001 versus BAA-835 group.

Amuc_1100 and P9 are key effector proteins for AKK’s effects on obesity and glucose metabolism. To understand why pAKK strains differ in alleviating MetS, we aligned these proteins across 8 AKK isolates and ATCC BAA-835. P9 identities ranged from 99.28–99.91% (nucleotide) and 99.59–100% (amino acid) (Supplementary Figures S1A,B). Amuc_1100 identities ranged from 97.69–99.37% (nucleotide) and 98.42–100.00% (amino acid) (Figure S2A-B). AKK2645 has five Amuc_1100 substitutions, none affecting core domain structure or function (Supplementary Figure S3A). The three-dimensional architecture of P9 was substantially conserved, and a subtle deviation at the C-terminal helix was attributed to a visualization artifact (Figure 7A). Despite pronounced sequence conservation among the tested strains, transcriptomic abundance displayed notable inter-strain heterogeneity. P9 protein levels were also strain-dependent, with AKK2645 showing markedly higher production than all other isolates—exceeding the lowest-producing strain by more than 10-fold (Figure 7B). AKK2645 demonstrated elevated transcript levels of Amuc_1100 relative to the other eight AKK (Supplementary Figure S3B). Transcript abundance correlated positively with MetS efficacy, with AKK2645 demonstrating superior weight control and glycemic improvement (Supplementary Figure S3C; Figure 7C). These findings collectively indicate that strain-dependent efficacy among AKK isolates may be partially driven by differential secretion or production of functional effector molecules. Moreover, the enhanced metabolic benefits conferred by AKK2645 may stem from transcriptional upregulation of P9 and Amuc_1100.

## 4 Discussion

Through systematic large-scale screening, this study presents the first direct evidence of substantial functional heterogeneity among pAKK strains in ameliorating MetS, with strain AKK2645 emerging as the most efficacious. This strain demonstrated enhanced efficacy in attenuating obesity, metabolic dysfunction, hepatic steatosis, and inflammation across multiple tissues. Collectively, these findings not only establish a potent postbiotic candidate for MetS but also extend the established paradigm of intraspecies functional heterogeneity—previously documented in probiotics (31–35)—to the postbiotic domain (36).

Previous investigations of AKK-derived postbiotics have primarily utilized a solitary reference strain (ATCC BAA-835) (17, 37, 38). In contrast, our comparative *in vitro* and *in vivo* analyses demonstrate that numerous pAKK isolates, with AKK2645 exhibiting particularly pronounced anti-adipogenic and anti-inflammatory effects, surpass this benchmark. This finding highlights the indispensable role of strain-specific characterization in the judicious development of postbiotic therapeutics (5, 39, 40).

The mechanistic basis for AKK2645’s enhanced therapeutic efficacy lies in its synergistic targeting of multiple pathophysiological pathways, a characteristic that distinguishes it from previously investigated interventions. For instance, while the pAKK strain Akk11 has been shown to enhance intestinal barrier integrity and regulate metabolism, it failed to significantly reduce body weight or serum TC levels (41). By contrast, AKK2645 administration resulted in an 11.41% decrease in body mass and a 21.99% reduction in serum TC. Furthermore, in addition to its established impacts on glucose and lipid metabolism, AKK2645 exhibits a distinctive immunomodulatory profile that distinguishes it from comparator strains and other inactivated probiotic formulations. Chronic low-grade inflammation constitutes a core pathological mechanism driving both the onset and progression of MetS (42, 43). Although pasteurized *Akkermansia muciniphila* ATCC BAA-835 has demonstrated efficacy in attenuating metabolic inflammation, its mechanism of action is primarily confined to the suppression of pro-inflammatory cytokines, with minimal impact on anti-inflammatory mediators such as IL-10 (44). In contrast, and in a manner divergent from heat-inactivated *Lactobacillus gasseri* (45), AKK2645 not only suppresses hepatic pro-inflammatory cytokines (IL-6 and IL-1β) but also demonstrably upregulates the expression of anti-inflammatory mediators (*Il4*, *Il10*, and *Foxp3*) within both white and brown adipose tissues (46, 47). This coordinated, multi-tissue induction of an anti-inflammatory environment likely underpins its superior comprehensive metabolic benefits, thereby establishing the mechanistic rationale for its augmented therapeutic efficacy (48, 49).

Administration of pasteurized AKK2645 elicited a profound remodeling of the gut microbiota in mice subjected to a HFD. This remodeling was characterized by the enrichment of multiple genera known for short-chain fatty acids (SCFAs) production, including *Akkermansia*, *Lactobacillus*, *Bifidobacterium*, and *Faecalibacterium*. Within this microbial community, *Faecalibacterium* and *Lactobacillus* are particularly noted for their significant capacity to generate butyrate and propionate (29). Intestinal SCFAs engage with the free fatty acid receptor 2 (FFAR2) and free fatty acid receptor 3(FFAR3) receptors expressed on L-cells, initiating intracellular signaling pathways that culminate in the secretion of GLP-1 and *Pyy* (50). There is evidence to suggest that butyrate may exert a more pronounced and enduring influence on L-cells differentiation and the sustained maintenance of secretory functions (51). Consequently, we hypothesize that the AKK2645-induced augmentation of SCFAs-producing bacteria potentiates endogenous intestinal SCFAs biosynthesis, thereby activating GLP-1-mediated signaling pathways. This proposed mechanism likely underlies the substantial upregulation of *Gcg* and *Glp-1r* expression documented in this investigation, as well as the observed concurrent amelioration of glucose tolerance. Definitive elucidation of the causal role of this signaling cascade in mediating the metabolic advantages associated with AKK2645 administration will necessitate future investigations employing targeted metabolomics for quantitative profiling of luminal SCFAs concentrations, alongside the utilization of selective receptor antagonists or gnotobiotic animal models.

Differential capacities for mucin utilization and immunomodulation have been documented among AKK strains (13, 36). AKK2645 may exhibit a distinct surface architecture, metabolic repertoire, or extracellular vesicular payload (38, 39). Considerable evidence has established that two representative effector proteins of *Akkermansia muciniphila*—Amuc_1100 and P9—play pivotal roles in host metabolic regulation. Amuc_1100, a conserved outer membrane protein, has been reported to engage TLR2 signaling, thereby modulating fatty acid metabolism and bile acid synthesis through TGR5/FXR pathways, enhancing intestinal barrier integrity, and influencing gut microbiota composition (52–54). P9, a secreted protein identified in AKK, has been shown to stimulate GLP-1 secretion from intestinal L cells via ICAM-2 binding (55). These observations collectively implicate both proteins as key mediators of the metabolic benefits attributed to AKK-derived postbiotics. In this study, strain AKK2645, harvested during logarithmic growth phase, exhibited a higher relative transcript abundance of Amuc_1100 compared to other isolates. Given that Amuc_1100 is a membrane protein, its elevated transcription in viable bacteria likely correlates with increased protein abundance, a characteristic potentially retained in pAKK powders. The P9 was also detected in the inactivated bacterial powders, and its concentration was quantified. It is noteworthy that across distinct AKK strains, P9 protein expression shows a consistent trend with observed phenotypic measures in animal models, including body weight, FBG, and AUC. Further investigation is warranted to determine whether the observed variations in protein expression directly precipitate the phenotypic alterations.

While this investigation furnishes robust evidence for the enhanced efficacy of AKK2645 in mitigating MetS, with this superiority demonstrably linked to heightened transcriptional and proteinaceous expression of P9 and Amuc_1100, we concede that several mechanistic lacunae necessitate further inquiry. Primarily, the molecular mechanisms underlying the differences in the expression of these effector proteins across different strains remain unclear. Future research could systematically elucidate the functional differences among different strains through whole-genome sequencing, comparative genomics analysis, and metabolomics studies of representative strains. Secondarily, the data from this study have not yet established a clear causal relationship. To address this, we will conduct recombinant protein supplementation, target gene knockout, and heterologous expression complementation experiments to verify whether P9 and Amuc_1100 are direct functional mediators in improving MetS. Through the precise delineation of these constraints, we endeavor to delineate a perspicuous trajectory for the future mechanistic dissection of strain-specific postbiotic efficacy.

Collectively, this work necessitates a paradigm shift in postbiotic development: from the use of arbitrary or single reference strains toward the evidence-based selection of specific, high-efficacy strains pasteurized AKK2645 emerges as a stable, safe. We have published the complete toxicological safety assessment report for this formulation (21). AKK2645 has obtained FDA GRAS certification and is the first and only applicant for New Food Raw Material status in China. A comprehensively effective postbiotic candidate with significant translational potential for metabolic syndrome. Future research employing comparative multi-omics can delineate the molecular determinants of AKK2645’s superiority, informing the rational design of even more targeted postbiotic formulations.

## Data Availability

The raw sequencing data have been deposited in the NCBI Sequence Read Archive (SRA) under BioProject accession number PRJNA1476887.
