# Supplementary material for: Unveiling functional heterogeneity in pasteurized Akkermansia muciniphila identifies AKK2645 as a superior postbiotic for metabolic syndrome
Source: Front Nutr. 2026 Aug 6;13:1908600. doi: 10.3389/fnut.2026.1908600 (PMC13490184; doi:10.3389/fnut.2026.1908600)
Supplement: Supplementary file 1 [file Table_1.docx]

Supplementary Material

# 1 Supplementary Tables

## 1.1 Table S1 Primers used for quantitative RT-PCR analysis.

**Mice**

| Gene | Forward primer (5’-3’) | Reverse primer (5’-3’) |
| --- | --- | --- |
| *fgfr4* | TCCATGACCGTCGTACACAAT | ATTTGACAGTATTCCCGGCAG |
| *glp-1r* | TCAGAGACGGTGCAGAAATG | CAGCTGACATTCACGAAGGA |
| *cyp7a1* | AGCAATGAAAGCAGCCTCTGA | TGATGCTATCTAGTACTGGCAGGT |
| *nr3c1* | CCGGGTCCCCAGGTAAAGA | TGTCCGGTAAAATAAGAGGCTTG |
| *shp* | TCTGCAGGTCGTCCGACTATTC | AGGCAGTGGCTGTGAGATGC |
| *hnf-4α* | GGTCAAGCTACGAGGACAGC | ATGTACTTGGCCCACTCGAC |
| *pepck* | CTGCATAACGGTCTGGACTTC | CAGCAACTGCCCGTACTCC |
| *il-4* | GGTCTCAACCCCCAGCTAGT | GCCGATGATCTCTCTCAAGTGAT |
| *il-10* | TGCTATGCTGCCTGCTCTTA | TCATTTCCGATAAGGCTTGG |
| *gcg-1* | CCGCCGTGCCCAAGA | CATCATGACGTTTGGCAATGTT |
| *glp-1r* | TCAGAGACGGTGCAGAAATG | CAGCTGACATTCACGAAGGA |
| *pyy* | ACGGTCGCAATGCTGCTAAT | GACATCTCTTTTTCCATACCGCT |
| *fgf15* | GCCATCAAGGACGTCAGCA | CTTCCTCCGAGTAGCGAATCAG |
| *g6pc1* | CGACTCGCTATCTCCAAGTGA | GTTGAACCAGTCTCCGACCA |
| *hsl* | CCAGCCTGAGGGCTTACTG | CTCCATTGACTGTGACATCTCG |
| *cpt* | ATGTATCGCCGCAAACTGGACC | CTCTGAGAGGTGCTGTAGCAAG |
| *ucp-1* | CTTTGCCTCACTCAGGATTGG | ACTGCCACACCTCCAGTCATT |
| *pgc-1α* | AGCCGTGACCACTGACAACGAG | GCTGCATGGTTCTGAGTGCTAAG |
| *ppar-α* | GGTCTCAACCCCCAGCTAGT | GCCGATGATCTCTCTCAAGTGAT |
| *foxp3* | ACCATGACTAGGGGCACTGTA | TCCACTCGCACAAAGCACTT |
| *β-actin* | CTGCGTTTTACACCCTTTCTTTG | GCCATGCCAATGTTGTCTCTTAT |

**Bacteria**

| Genus | Name | Sequence |
| --- | --- | --- |
| *Bifidobacterium* | BIF-D9f | CTTACTTCGCCTTCTTTGCTCCRTAC |
|  | BIF-D9R | AGAAGTCCAAGACTTTGGCCCTGA |
| *Lactobacillus* | LactoF | TGGAAACAGRTGCTAATACCG |
|  | LactoR | GTCCATTGTGGAAGATTCCC |
| *Akkermansia* | AM1 | CAGCACGTGAAGGTGGGGAC |
|  | AM2 | CCTTGCGGTTGGCTTCAGAT |

## 1.2 Table s2. Effects of Different pAKK  on TG Content in a HepG2 Cell Model with Lipid Accumulation.

| **Group** | **TG inhibition rate（%）** | **Group** | **TG inhibition rate（%）** |
| --- | --- | --- | --- |
| Con | **-** | AKK 2603 | 6.52 |
| OA | **-** | AKK 2604 | 0.00 |
| BAA-835 | 32.61 | AKK 2605 | 28.26 |
| AKK 2583 | 0.00 | AKK 2606 | 26.09 |
| AKK 2584 | 19.57 | AKK 2607 | 10.87 |
| AKK 2585 | 0.00 | AKK 2609 | 45.65 |
| AKK 2586 | 10.87 | AKK 2610 | 0.00 |
| AKK 2587 | 39.13 | AKK 2618 | 26.09 |
| AKK 2588 | 15.22 | AKK 2625 | 45.65 |
| AKK 2589 | 10.87 | AKK 2635 | 19.57 |
| AKK 2590 | 15.22 | AKK 2638 | 39.13 |
| AKK 2591 | 19.57 | AKK 2639 | 39.13 |
| AKK 2592 | 17.39 | AKK 2642 | 41.30 |
| AKK 2593 | 41.30 | AKK 2643 | 36.96 |
| AKK 2594 | 17.39 | AKK 2645 | 58.70 |
| AKK 2595 | 0.00 | AKK 4071 | 0.00 |
| AKK 2596 | 4.35 | AKK 4072 | 45.65 |
| AKK 2597 | 15.22 | AKK 4080 | 0.00 |
| AKK 2598 | 13.04 | AKK 4081 | 0.00 |
| AKK 2599 | 36.96 | AKK 4082 | 26.09 |
| AKK 2600 | 26.09 | AKK 4083 | 36.96 |
| AKK 2601 | 45.65 | AKK 4087 | 0.00 |
| AKK 2602 | 52.17 |  |  |

## 1.3 Figure s1 Sequence alignment of the DNA and protein sequences of the Amuc_1100 gene from AKK strains. (A) DNA sequence alignment of the Amuc_1100 gene from nine AKK strains. (B) Protein sequence alignment of Amuc_1100 from nine AKK strains.

## 1.4 Figure s2 Sequence alignment of the DNA and protein sequences of the P9 gene from AKK strains. (A) DNA sequence alignment of the P9 gene from nine AKK strains. (B) Protein sequence alignment of P9 from nine AKK strains.

## 1.5 Figure s3 Sequence features, transcriptional abundance, and functional correlations of Amuc_1100 proteins. (A) Schematic representation of amino acid mutations in P9 between the AKK2645 strain and reference strains. (B) Relative transcriptional levels of P9 across different strains. (C)Heatmap showing correlations between transcript levels of the P9 proteins and key phenotypic indicators.Data are presented as mean ± SD.
